# Chemotherapy-Naïve Jejunal Lymphoma Fistulising With the Urinary Bladder

**DOI:** 10.7759/cureus.93521

**Published:** 2025-09-29

**Authors:** Riddhima Dubhashi, Sankar Subramaniam

**Affiliations:** 1 Surgery, Dr. Vaishampayan Memorial Government Medical College and Government Hospital Solapur, Solapur, IND; 2 Surgical Gastroenterology, Sri Ramachandra Institute of Higher Education and Research, Chennai, IND

**Keywords:** crohns disease, enterovesical fistula, jejunal perforation, non hodgkin's lymphoma, primary jejunal lymphoma

## Abstract

An elderly male was diagnosed with Crohn’s disease empirically elsewhere and put on mesalamine tablets. A few months later, he developed acute dysuria and bile-stained urine. Computerized tomography revealed a contained jejunal perforation with a phlegmonous collection eroding into the dome of the urinary bladder with multiple intact tablets within it. At laparotomy, the affected segment of the jejunum and dome of the bladder were resected en bloc and intact mesalamine tablets were retrieved. Appropriate reconstruction was done. Histopathology examination confirmed it to be diffuse large B-cell lymphoma. Primary jejunal lymphoma is a rare occurrence with non-specific clinical presentation, posing diagnostic challenges. Perforation of chemotherapy-naïve lymphoma is uncommon and fistulizing into the bladder is even rarer.

## Introduction

Primary gastrointestinal lymphomas are a rare occurrence, constituting 5-10% of gastrointestinal tumors. Perforation is a life-threatening complication of lymphomas involving the gastrointestinal tract, commonly associated with aggressive variants of lymphoma. It may occur after initiation of chemotherapy or may even be seen as the initial presentation. The clinical presentation is non-specific, posing diagnostic dilemmas. The presenting case highlights the rare complicated presentation of a primary jejunal lymphoma, requiring a high index of suspicion [1].

## Case presentation

**Figure 1:**
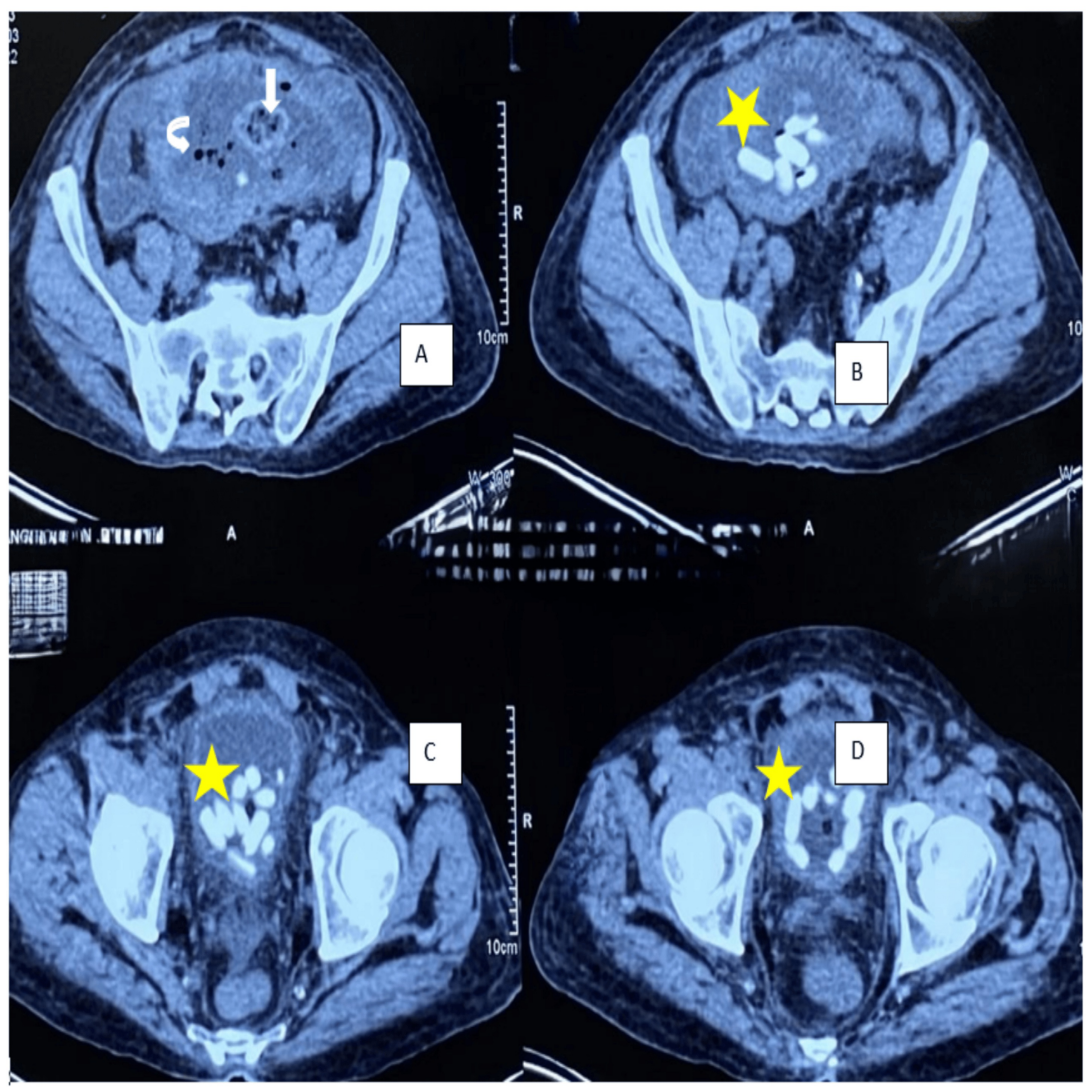
Contrast-enhanced CT scan of the abdomen and pelvis A-D: Axial post-contrast abdomen in venous phase at the level of the pelvis shows well-defined thick-walled abscess collection in the lower abdomen (white arrow) with multiple air pockets (down arrow) and trapped jejunal loops within. Multiple hyperdense tubular foci are seen within the urinary bladder (asterisk) likely representing retained non-absorbed tablets, suggesting the possibility of enterovesical fistula with sealed off abscess collection.

**Figure 2:**
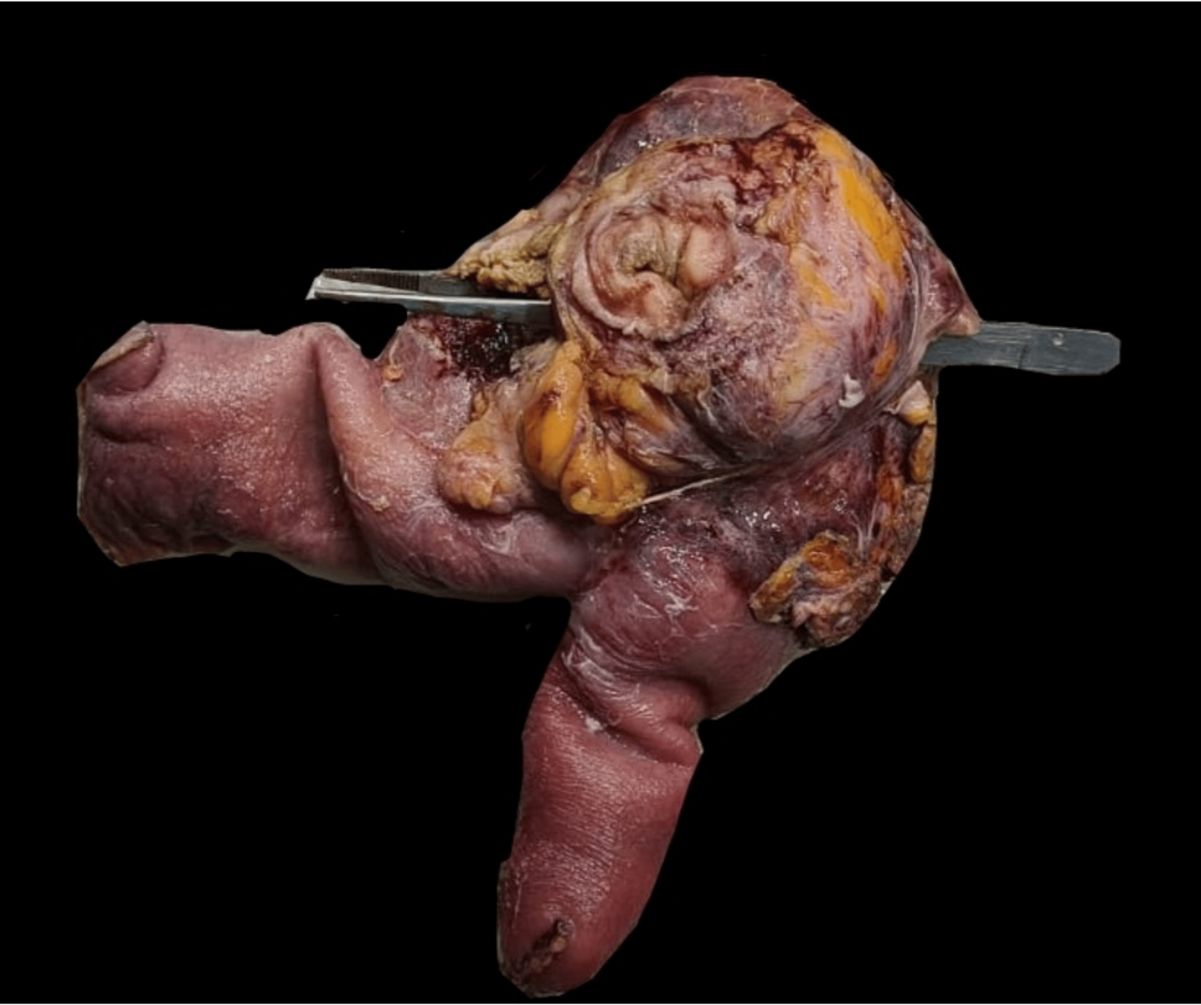
Resected specimen: phlegmon composed of entero-vesical fistula, jejunum and a cuff of urinary bladder.

**Figure 3:**
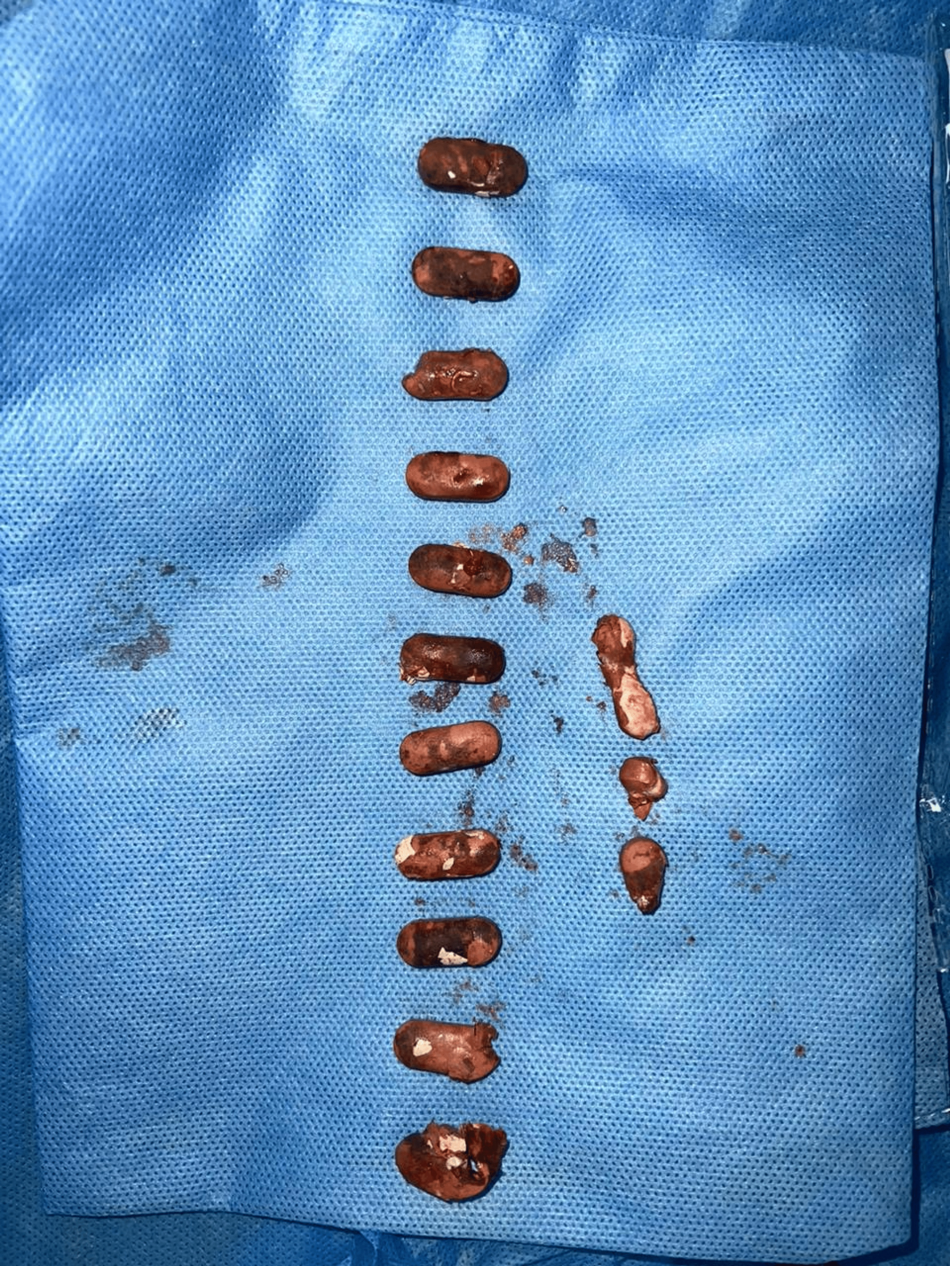
Intact mesalamine tablets retrieved from the urinary bladder

An elderly male in his mid-60s presented with haematuria and dysuria for three days. He had a history of hospitalization at an outside hospital five months ago for generalized weakness and easy fatiguability and was diagnosed with dimorphic (iron + vitamin B12 deficiency) anemia. He was treated with ferric carboxy maltose and vitamin B12 and folate supplementation. He underwent upper gastrointestinal endoscopy to evaluate the cause of the anemia which revealed two duodenal ulcers. Antral mucosal biopsy was suggestive of H. pylori-induced chronic gastritis and the antral mucosa was positive for rapid urease test. A diagnosis of H. pylori infection was made, for which he was treated. He also underwent a contrast-enhanced computed tomography (CT) scan of abdomen during that admission for any extraluminal source of bleeding, which reported mild jejunitis. At time of discharge from that hospital, he was given oral iron and vitamin B12 supplements to continue for one month. He was readmitted at that hospital three months later, with repeated complaints of easy fatiguability, weakness and significant weight loss. Blood Investigations revealed iron deficiency anemia, for which treatment was instituted. Repeat gastrointestinal endoscopy revealed healed duodenal ulcers. CT enterography and contrast-enhanced CT of the abdomen showed mild wall thickening of the jejunum with a few hyper-enhancing foci of 3-10 mm in the jejunum. Mesenteric lymphadenopathy (13x9mm) was noted in the left hypochondrium and left lumbar regions. Fecal calprotectin was 237 mcg/dL. A decision was made to do a single balloon enteroscopy to visualize the jejunal loops and look for features suggestive of Crohn’s disease. It revealed multiple erosions and aphthous ulcers in the jejunum and biopsy of the lesions showed scattered scanty lymphoplasmic infiltrates with no confirmatory signs of Crohn’s disease or malignancy. An empirical diagnosis of Crohn’s disease was made considering high fecal calprotectin, CT report and blood tests. He was started on tab mesalamine. Two months later, he developed haematuria and dysuria and then presented to our hospital. His ultrasonography abdomen at admission revealed diffuse thickening of the bladder wall with multiple calculi (10-15) with suspicious discontinuity in the dome of the bladder and a suspicious collection of 6.5 x 6.2 cm in the supra-vesical region communicating with the urinary bladder. Contrast-enhanced CT of abdomen showed a loculated well-defined fluid collection (8.8 x 12.8 x 8.1 cm) with particulate matter, air pockets and large enteroliths in the pelvic region in midline. Pelvic small bowel loops/distal jejunal and proximal ileal loops adherent to the collection. Fistulous communication of 1.5 x 1.3 cm was noted between the lower aspect of the collection and the urinary bladder, with multiple enteroliths in the bladder (Figure 1). On the basis of these investigations and his presenting complaints, a decision was made to operate on the patient and he was posted for an exploratory laparotomy. Patient underwent exploratory laparotomy, which revealed a fistulous communication between the jejunal loops and the dome of the urinary bladder. The enterovesical fistula, along with the mass and affected segment of jejunum, was excised (Figure 2). Intact mesalamine tablets were retrieved from the urinary bladder (Figure 3). The excised phlegmon along with fistula tract, jejunal loops and cuff of the urinary bladder was sent for histopathological examination. The histopathological examination revealed a diffuse large B-cell lymphoma (DLBCL) of the jejunum.

Table 1 contains the laboratory values of the patient when he was diagnosed with anemia and the fecal calprotectin levels that were high and hence the diagnosis of Crohn's disease was made at an outside hospital and he was started empirically on tablet mesalamine.

**Table 1:** Table showing the patient's laboratory investigations

INVESTIGATION	VALUE	NORMAL RANGE
Haemoglobin (in May 2023)	6.5 g/dl	12-15 g/dl
Haemoglobin (in August 2023)	7.1 g/dl	12-15 g/dl
Faecal Calprotectin	237 ug/g	<100 ug/g

## Discussion

Entero-vesical fistula formation is a rare complication that is observed secondary to malignancies, trauma, radiation and inflammatory bowel disease [1]. The morbidity associated with this condition significantly affects one’s quality of life. This case is a rare presentation of a fistula occurring in a patient with B-cell jejunal lymphoma, not treated with chemotherapy. DLBCL is a neoplasm of large transformed B cells that mimics centroblasts/immunoblasts, with a significant proliferation fraction of >40-90%, with 40% cases presenting at extra-nodal sites [2]. Jejunum, as compared to ileum, has less lymphoid tissue, and is a rare site for occurrence of primary DLBCL. The presenting features are often non-specific, which pose a diagnostic dilemma. Vien et al. [3] report a case initially diagnosed as viral gastroenteritis, with the diagnosis of lymphoma being made on biopsy reports one month later. A case of DLBCL with small intestinal perforation has been reported in an elderly female, presenting with acute abdomen [4]. Warsinggih et al. [5] reported a similar case presenting with generalized abdominal pain, fever and significant weight loss. Forty percent of cases have obstruction as the presenting feature [6]. H. pylori infection as a risk factor has been reported in several studies [7,8]. Our case had presented initially with abdominal pain, haematuria, dysuria, and weight loss. Investigations had pointed towards anaemia and H. pylori-induced gastritis. Initial CT imaging findings had led the then-treating clinician to an initial diagnosis of Crohn’s disease. The process of fistula formation is a chronic one, with the lesions gradually invading the serosa and perforating into the mesentery, with confined abscesses or fistulous communications with adjacent organs. Several cases of DLBCL of jejunum often have had a delayed presentation with complications [9]. Multiple skip lesions on imaging, along with features of bowel perforation, can subject the case to emergency laparotomy, wherein the diagnosis of DLBCL can be arrived at, only after histopathological examination. Abdominal tomography is a reliable tool for the diagnosis of entero-vesical fistula. Primary resection anastomosis of the affected bowel segment, with primary repair of the urinary bladder, is the treatment [10].

## Conclusions

Primary jejunal extra-nodal lymphomas are very rare and usually present with non-specific symptoms, which can delay the diagnosis. Some cases may even look like Crohn’s disease on initial evaluation. Surgery with chemotherapy is the mainstay of treatment. Perforations are mostly seen after chemotherapy, but in chemotherapy-naïve patients they are extremely uncommon, and extension into the urinary bladder is even rarer. This makes the present case unusual and highlights the importance of considering lymphoma in such difficult clinical situations.
